# Genetic changes are introduced by repeated exposure of *Salmonella* spiked in low water activity and high fat matrix to heat

**DOI:** 10.1038/s41598-021-87330-8

**Published:** 2021-04-14

**Authors:** Leen Baert, Johan Gimonet, Caroline Barretto, Coralie Fournier, Balamurugan Jagadeesan

**Affiliations:** 1 Nestlé Research, Vers-Chez-les-Blanc 26, 1000 Lausanne, Switzerland; 2 Nestlé Research, EPFL Innovation Park, 1015 Lausanne, Switzerland

**Keywords:** Microbiology, Microbial genetics, Bacterial genetics

## Abstract

WGS is used to define if isolates are “in” or “out” of an outbreak and/or microbial root cause investigation. No threshold of genetic differences is fixed and the conclusions on similarity between isolates are mainly based on the knowledge generated from previous outbreak investigations and reported mutation rates. Mutation rates in *Salmonella* when exposed to food processing conditions are lacking. Thus, in this study, the ability of heat and dry stress to cause genetic changes in two *Salmonella* serotypes frequently isolated from low moisture foods was investigated. *S*. enterica serovars *S*. Agona ATCC 51,957 and *S*. Mbandaka NCTC 7892 (ATCC 51,958) were repeatedly exposed to heat (90 °C for 5 min) in a low water activity and high fat matrix. No increased fitness of the strains was observed after 10 repeated heat treatments. However, genetic changes were introduced and the number of genetic differences increased with every heat treatment cycle. The genetic changes appeared randomly in the genome and were responsible for a population of diverse isolates with 0 to 28 allelic differences (0 to 38 SNPs) between them. This knowledge is key to interpret WGS results for source tracking investigations as part of a root cause analysis in a contamination event as isolates are exposed to stress conditions.

## Introduction

*Salmonella* is a major cause of gastroenteritis in humans. In the USA, nontyphoidal *Salmonella* is the second major cause (11%) of foodborne illness identified after Norovirus (58%) while the leading cause of hospitalization is nontyphoidal *Salmonella* infections (35%)^1^. *Salmonella* transmission is serotype dependent^2^ and involves diverse foods like egg based products, ready-to-eat food, meat and fresh produce^3–5^. Low moisture foods such as powdered infant formula, peanut butter and chocolate have also been implicated in salmonellosis outbreaks^6^. In the public health context, identification of the source of a foodborne illness and outbreak is crucial to remove the potential food source from the market, if the outbreak is still ongoing, and to prevent recurrent issues. Along with the epidemiological investigation, subtyping tools are essential in identifying the source of a foodborne outbreak. Classical subtyping methods based on phenotypic characteristics (e.g. serotyping, phage typing) or genomic features (Pulsed Field Gel Electrophoresis (PFGE), Multi-Locus Variable number tandem repeat Analysis (MLVA) are increasingly replaced by whole genome sequencing (WGS) analysis^4,7–9^. Following the lead made by the authorities on the use of WGS in the public health sphere, the technology is increasingly being adopted by the food industry primarily for pathogen source tracking investigations as part of a root cause analysis in a contamination event^10,11^. Such an investigation can include isolates originating from a food processing plant that might have been exposed to environmental stress conditions over time which is different to the investigation of a foodborne illness that most often involves isolates limited to the duration of an outbreak.

WGS analysis allows the comparison of isolates up to the nucleotide level. By using high quality Single Nucleotide Polymorphisms (hqSNP) analysis, coding and non-coding regions are compared between isolates and quality filtered nucleotide differences are identified. An alternative approach is the use of core genome (cg) or whole genome (wg) Multi Locus Sequence Typing (MLST) where differences within a set of loci are compared between isolates. Allelic differences obtained by wgMLST showed similar ranges as hqSNP differences among *Listeria monocytogenes* isolates^12^. Although hqSNP analysis takes into consideration the complete genome in the analysis, hqSNP and wgMLST have been shown to be equally discriminatory to determine strain relatedness and provide epidemiologically concordant results in outbreak investigations^13,14^.

WGS results interpretation depends on pairwise SNP distances between isolates, bootstrap support of the branches in a phylogenetic tree and the tree topology^15^. Among these three parameters, genetic distances estimated as SNP and in case of cg/wgMLST analysis as allelic differences, often forms the initial basis to determine the similarity between isolates. Interpretation of SNP/ allelic values to conclude on the similarity of isolates can be challenging as absolute thresholds do not exist or it is not feasible to prescribe one. The prevailing knowledge on the genetic distances to determine the similarity between isolates is mostly derived from previous outbreak investigations and reported mutation rates. In a cross-sectional study, Wang et al.^16^ investigated 6,351 *Salmonella* isolates originating from 2,196 facilities and concluded that isolates from within a facility will have fewer (< 20) SNP differences (probability = 0.66), though larger differences could also occur. The outbreak investigation of *S*. Agona infections among infants in 2017 indicated that the outbreak isolates clustered within a maximum distance of 26 SNPs and these isolates originated from cases with gastrointestinal symptoms between April and December of 2017^17^. *Salmonella* mutation rates of 9.3 × 10^–8^ per nucleotide/year for the accumulation of core SNPS (or 0.44 SNPs per genome/year) calculated for *S*. Agona^18^ and 2.2 × 10^–7^ per site/year (or 1.01 SNPs per genome/year) calculated for *S*. Enteritidis^19^ are reported. An extrapolation of these mutation rates does not correlate with the number of SNP differences observed between the outbreak S. Agona isolates within a time period of 8 months, indicating the potential role of other factors. However, there is limited information on the *Salmonella* evolution rate in the farm to fork continuum which is essential to interpret WGS results of isolates of food origin and its associated environment. Though WGS analysis of longitudinal set of *Salmonella* isolates recovered from a single facility might provide insights into the mutation rates, such studies are generally lacking and even more it is difficult to link the impact of specific environmental or food process conditions on the genetic changes. In this context, the objective of this study was to evaluate the ability of simulated sublethal food processing conditions to induce genetic changes in *Salmonella* as to the best of our knowledge this phenomenon has not been studied. Two model serotypes frequently associated with low moisture foods were used in this study. *S*. Agona has been associated with foods such as aniseed-containing herbal tea^20^, infant milk products^17^ and cereals^21^ while *S.* Mbandaka has been associated with cereals and is a commonly found *Salmonella* serovar^22,23^. The strains were repeatedly exposed to heat in a low moisture matrix to assess its impact to introduce genetic changes. In addition, phenotypic parameters namely, log reduction, lag time and sublethal injury levels were studied to evaluate if changes in fitness could be observed and if so to potentially correlate with the introduced nucleotide changes.

## Materials and methods

### Experimental design

**Figure 1:**
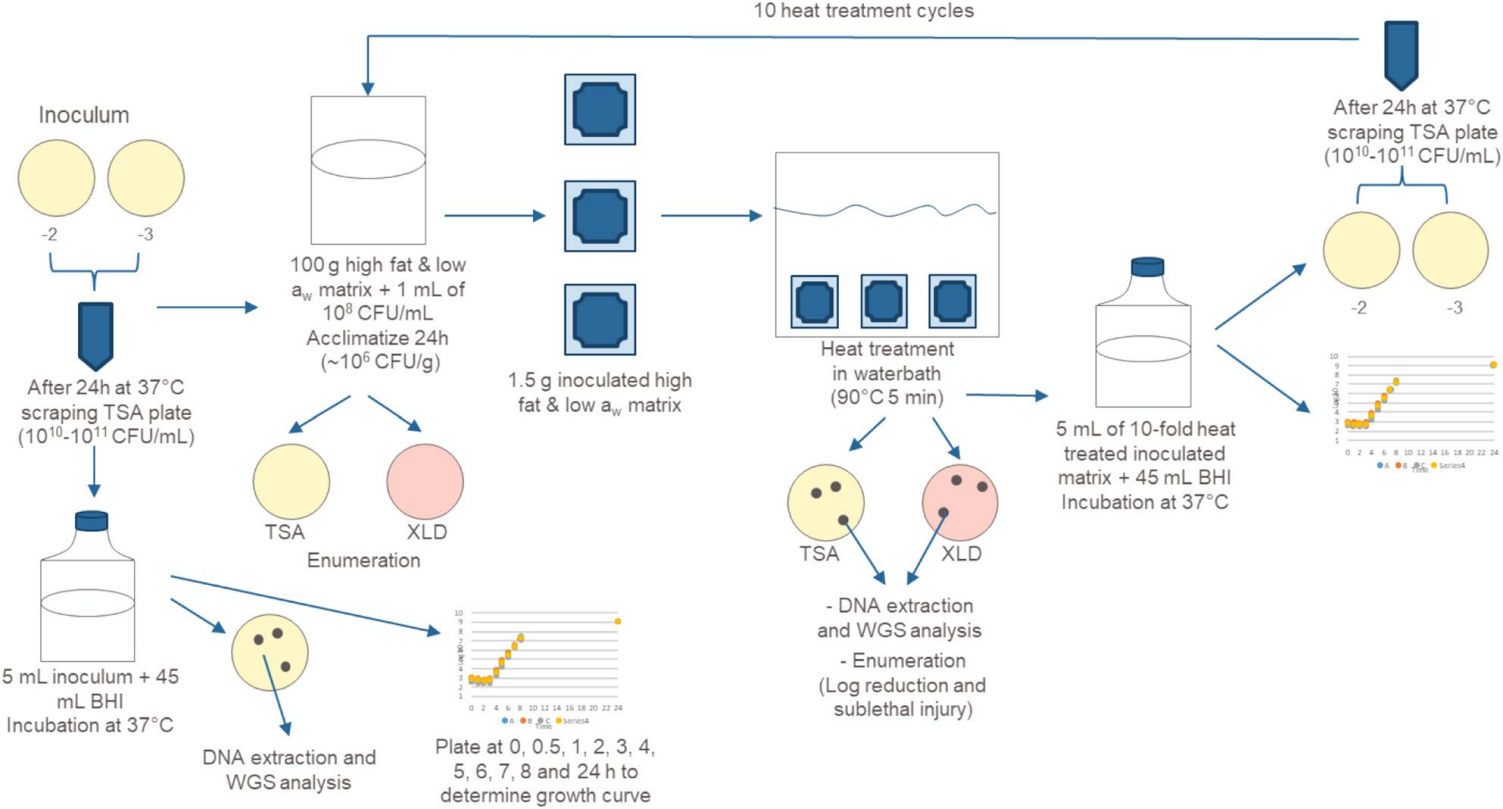
Overall experimental design to assess the impact of simulated food process conditions on the mutation rate in *Salmonella*.

The overall experimental procedure is outlined in Fig. 1. Previously, we have shown that a maximum of only 1 SNP was introduced after 10 sub-culturing steps in TSA/ Columbia agar (37 °C, 24 ± 2 h) for *Salmonella enterica* serovars (Tennessee, Hadar, Typhimurium and Enteritidis)^24^. While mutations can be caused by sub-culturing steps, based on our previous findings, this is less likely to happen and thus we premised that mutations observed following this protocol would largely be induced by exposure to stress conditions.

### Inoculum preparation for the first heat treatment cycle

*Salmonella enterica* Agona (ATCC 51,957) and *Salmonella enterica* Mbandaka (NCTC 7892 = ATCC 51,958)) stored on cryobeads (TSC, Lancashire, UK) at -80 °C were streaked on Trypticase Soy agar (TSA) (ThermoScientific Oxoid, Hampshire, UK) to obtain single colonies. After 24 h incubation at 37 °C, one colony was inoculated into 4 mL of Brain Heart Infusion (BHI) broth (ThermoScientific Oxoid, Hampshire, UK) and incubated at 37 °C for 24 h. Subsequently, a tenfold serial dilution in Tryptone Salt (TS) (ThermoScientific Oxoid, Hampshire, UK) was prepared and plated on TSA (37 °C, 24 h). The lawn growth obtained from 10^–2^ and 10^–3^ dilutions were scraped off using 2 mL of TS to obtain the inoculum. The inoculum (approximately 10^10^–10^11^ CFU/mL) was stored at 4 °C prior to the inoculation of the matrix.

### Inoculation of low water activity and high fat matrix (hereinafter matrix)

The inoculum was diluted 100-times in TS and 1 mL was inoculated in 200 µL droplets to 100 g of sterile (irradiated Synergy Health, Däniken, CH) dry matrix in a stomacher bag (1% inoculum level to obtain a target level approximately 10^6^ CFU/g). Afterwards, the bag was massaged by hand until no clumps were observed anymore. Each time 3 replicates were analyzed after inoculation to ensure the inoculation was done homogeneously. The standard deviations for each of the 10 inoculated matrices was on average 0.14 and 0.17 log cfu/g for *S*. Agona and *S*. Mbandaka, respectively, showing the inoculation was homogenous. The matrix consisted of dried digested animal byproducts, an ingredient used for the coating of dry pet food, with a composition of 3.1% moisture, 60% protein and 25% fat content. The a_w_ of the non-inocuated matrix was 0.208. The inoculated matrix was hermetically closed in an aluminum bag (Vacopack, Jehud, Israel) and stabilized in an incubator at 25 °C during 24 h following which the a_w_ increased to 0.275.

### Heat treatment of the spiked matrix

Thermal cells as described by Rachon et al.^25^ were used to carry out the heat treatments. For each replicate, 1.5 g of inoculated matrix was transferred to a thermal cell (0.8 mm deep,48 mm diameter). Three replicates were prepared. The control thermal cell, containing 1.5 g non-inoculated matrix, had an incorporated built-in platinum thermocouples (Pt 100) designed and supplied by Nestlé Research (Lausanne, Switzerland). In each trial, temperature profiling was conducted and the core temperature of samples were recorded using a data logger (PicoLog TC-08; St Neots, UK). The 3 replicates with inoculated matrix and the control thermal cells were immersed in a water bath at 90 °C for 5 min. After the heat treatment, the thermal cells were returned to room temperature using a water bath (25 °C, 1 min).

### Enumeration on selective (XLD) and non-selective (TSA) medium

From the thermal cells, one g of heat-treated matrix was transferred to a stomacher bag and tenfold serial dilutions were prepared in TS to carry out the enumeration on TSA and Xylose Lysine Deoxycholate (XLD) (ThermoScientific Oxoid, Hampshire, UK) agar (24 h incubation at 37 °C). The difference in counts on TSA before and subsequent to the heat treatment were used to calculate the log reduction caused by each heat treatment cycle. The counts (CFU/g) on XLD, and TSA were used to calculate sublethal injury levels after each heat treatment cycle using the following formula: [(Count on TSA) – (Count on XLD)]/ (Count on TSA).

Enumeration of the inoculum level (*S*. Agona and *S*. Mbandaka) was also performed on TSA and XLD before it was used to spike the matrix.

### Determination of lag time and inoculum preparation for 10 consecutive heat treatment cycles

A growth curve of the inoculum was determined by adding 5 mL of the 10^–7^ dilution from the inoculum (approximately 10^10^–10^11^ CFU/mL) to 45 mL of preheated (37 °C) BHI broth. Enumeration was carried out on TSA at 0, 0.5, 1, 2, 3, 4, 5, 6, 7, 8 and 24 h to determine the growth curves and to calculate the lag phase of the inoculum using ComBase (A Web Resource for Quantitative and Predictive Food Microbiology. University of Tasmania; USDA Agricultural Research Service. https://data.nal.usda.gov/dataset/combase-web-resource-quantitative-and-predictive-food-microbiology).

Following the heat treatment, 5 mL of tenfold dilution from each sample was added to 45 mL of preheated (37 °C) BHI. Enumeration was carried out on TSA at 0, 0.5, 1, 2, 3, 4, 5, 6, 7, 8 and 24 h to determine growth curves and to estimate the lag phase by ComBase. The inoculum for the next round of heat treatment was prepared by growing a lawn culture of the 10^–2^ and 10^–3^ dilutions from a single inoculated sample on TSA (37 °C, 24 h) and subsequently following the procedure described above. This new inoculum was used to spike a new batch of matrix for the next cycle of heat treatment cycle. In total, 10 consecutive heat treatment cycles were performed on the two strains of *Salmonellae*. For each heat treatment cycle, 3 colonies picked from TSA and XLD, where possible, were sequenced from the inoculum and the 3 replicates of the heat treated matrix.

### Statistical analysis

A global p-value (ANOVA) as well as pairwise comparisons (post-hoc comparison without comparisons for multiplicity of tests) was calculated with R version 3.6.1 (R: A language and environment for statistical computing. R Core Team. R Foundation for Statistical Computing, Vienna, Austria,2019, ISBN 3–900,051-07–0, URL http://www.R-project.org/) for the log reduction on TSA and the lag time.

### WGS analysis

Three individual colonies from TSA and XLD respectively isolated from the inoculum and heat-treated samples were inoculated into 4 mL of BHI broth and incubated at 37 °C for 6 h. After incubation, an aliquot (one mL) of BHI was centrifuged at 5000 × g, 5 min. The pellet was store at -20 °C until the DNA extraction was performed according to Gimonet et al.^26^ and sequenced with HiSeq as described by Portmann et al.^24^. Data are publicly available under the PRJNA698748 BioProject.

In total, 160 isolates were sequenced for *S*. Agona (cycle 1–4: 12 isolates; cycle 5: 19 isolates; cycle: 6–7: 18 isolates; cycle 8–9: 21 isolates; cycle 10: 16 isolates). For *S*. Mbandaka, 210 isolates were sequenced (cycle 1–10: 21 isolates/cycle).

For *S*. Mbandaka, at least 3 colonies were present on XLD for all cycles but for *S*. Agona for cycles 5, 6, 7 and 10, less colonies were taken since not always 3 colonies for each replicate were available and no colonies were taken for cycles 1 to 4.

BioNumerics v7.6.3 (https://www.applied-maths.com/bionumerics) was used to carry out the wgMLST analysis. Assembly-free and assembly-based allele calling were used to calculate the allelic differences using the default settings. The *Salmonella* scheme consists of 15,874 loci. A minimum spanning tree (MST) was created to illustrate the relatedness between all sequenced isolates for *S.* Agona and *S*. Mbandaka.

High quality SNP (hqSNP) pipeline developed by the Center for Food Safety and Applied Nutrition (CFSAN) at the U.S. Food and Drug Administration (CFSAN SNP Pipeline v.1.0.1) was used for SNP calling on the isolates^27^.

Maximum-likelihood phylogenetic tree were built with GARLI (Version 2.01.1067^28^) on the SNP analysis results.

### Identification of genes in which genetic changes were observed for S. Agona and S. Mbandaka

Basic Local Alignment Search Tool (Blastn version 2.4.0 + ^29^) was used to identify the genomic regions containing a SNP comparing the concerned nucleotide region with the ATCC strains. Accession numbers of the ATCC strains are NZ_CP019183 for *S.* Mbandaka and NZ_AOZX01000005, NZ_AOZX01000007 NZ_AOZX01000011, NZ_AOZX010000636, NZ_AOZX01000039, NZ_AOZX01000043 and NZ_AOZX01000064 for *S.* Agona. SNPs were investigated that occurred at least 10 times along all *S*. Agona and *S*. Mbandaka isolates respectively.

Visualization of the genomic regions was done using Artemis Comparison Tool (ACT)^30^.

## Results

### Phenotypic characteristics following exposure of S. Agona and S. Mbandaka to heat and dry stress

**Figure 2:**
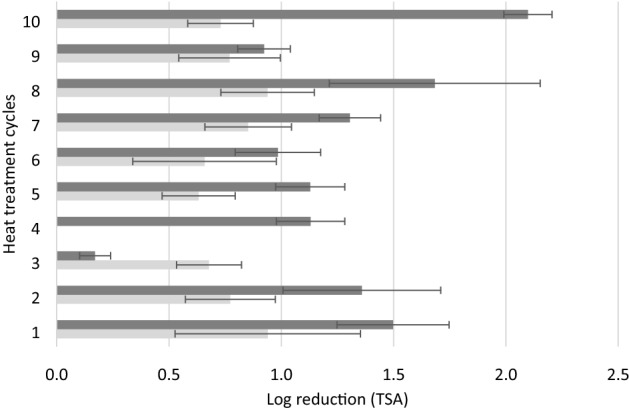
Log reduction of *S.* Agona (dark grey bars) and *S*. Mbandaka (light grey bars) determined from TSA counts (log CFU/mL) after heat treatment cycles (1 to 10) in a dry matrix (initial level approximately 10^6^ CFU/g). Error bars represent the standard deviation from three replicates.

The reduction in counts (CFU/g) by repeated heat treatments (90 °C for 5 min) in an inoculated low water activity, high fat matrix are presented in Fig. 2. Between 0.2 and 2.1 log reduction was obtained in the case of *S*. Agona. The 3^rd^ heat treatment cycle resulted in a markedly lower log reduction compared to the other cycles. In contrast, the 10^th^ cycle caused a significantly higher log reduction compared to the 9 previous heat treatment cycles. Overall, the log reduction of S. Mbandaka was similar (P > 0.05) among the 10 heat treatment cycles, except for cycle 4 where no log reduction was observed. The reduction levels were consistent for S. Mbandaka after the 4^th^ heat treatment cycle on TSA but 1.5 ± 0.4 log reduction was observed on the selective medium XLD (data not shown).

**Figure 3:**
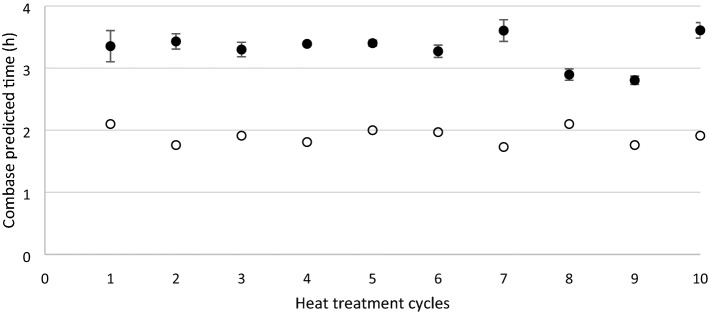
Predicted lag time of *S*. Agona after repeated (1 to 10) heat treatment cycles in a dry matrix. The lag time of the inoculum (blank circles) and after heat treatment (black circles) of *S*. Agona is illustrated. Error bars represent the standard deviation from three replicates.

The average % of sublethal injury levels before and after heat treatment are mentioned in Table 1. Sublethal injury levels varied in the inoculated matrix (before heat treatment) for both *Salmonella* strains. After heat treatment, > 98% of the surviving cells were sublethally injured and the variability between replicates was significantly lower.

**Table 1:** Sublethal injury (%) calculated from the count obtained from non-selective (TSA) and selective (XLD) plating.

	Before heat treatment (n = 10^a^)		After heat treatment (n = 10^a^)	
	Average	SD	Average	SD
*S.* Agona	24.8	28.1	99.3	1.8
*S*. Mbandaka	52.9	21.3	98.6	1.7

^a^Counts determined from 10 heat treatment cycles; each cycle included counts of the inoculated matrix from 3 replicates before the heat treatment and after heat treatment.

**Figure 4:**
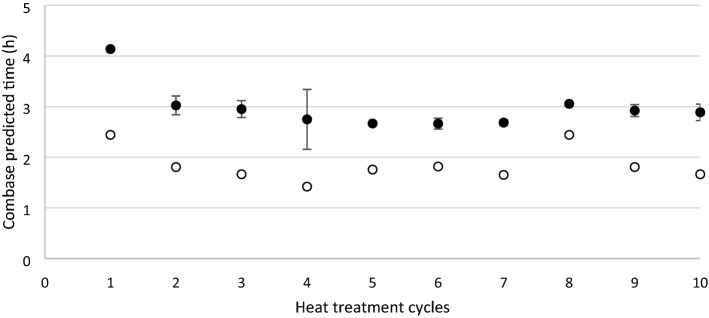
Predicted lag time of *S*. Mbandaka after repeated (1 to 10) heat treatment cycles in a dry matrix. The lag time of the inoculum (blank circles) and after heat treatment (black circles) of *S*. Mbandaka is illustrated. Error bars represent the standard deviation from three replicates.

Additionally, the lag time was calculated for (i) the inoculum that was used to spike the matrix and (ii) subsequent to the heat treatment of the spiked matrix. The lag time of the inoculum was stable along the 10 cycles (Figs. 3 and 4) and was significantly lower (P < 0.05) in comparison with the lag times of both strains after the heat treatment. The lag time of *S*. Agona after heat treatment was significantly lower (P < 0.05) after cycle 8 and 9 compared to the other cycles (Fig. 3). The lag time of *S.* Mbandaka from the first cycle was significantly higher (P < 0.05) compared to the 9 subsequent cycles (Fig. 4).

### Genotypic characteristics following exposure of S. Agona and S. Mbandaka to heat and dry stress

For each cycle, three colonies from (i) the inoculum used to spike the matrix plated on TSA, three colonies from the matrix after heat treatment plated on TSA and (iii) three colonies from the matrix after heat treatment plated on XLD, if available, were sequenced and analyzed by wgMLST and SNP analysis. Sequence data passed the minimum quality criteria (passing the “per base sequence quality” FASTQC threshold FastQC software (v0.11.5), ≥ 30 average coverage, < 200 contigs, de novo assembly sequence length ~ 4.7–4.8 million bp, 95% of the *Salmonella* core genome present).

**Figure 5:**
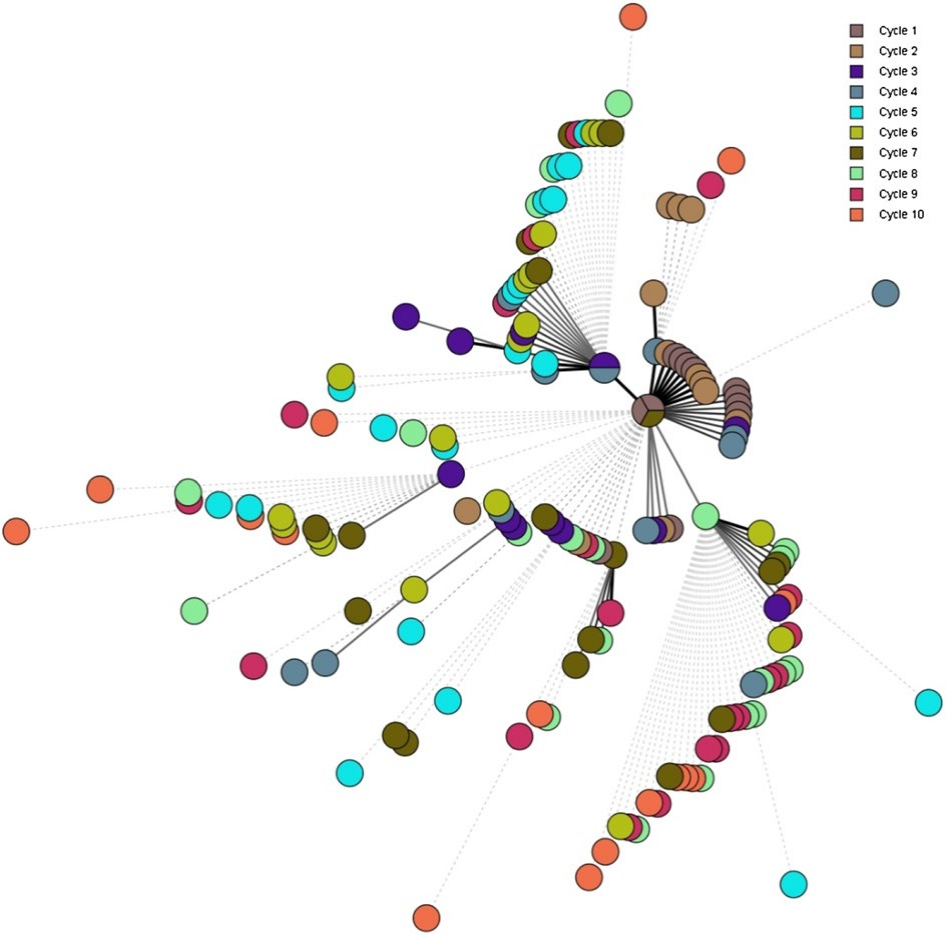
Minimum Spanning Tree of wgMLST analysis of *S.* Agona. Each circle represents 1 isolate and the colors illustrate the heat treatment cycle.

**Figure 6:**
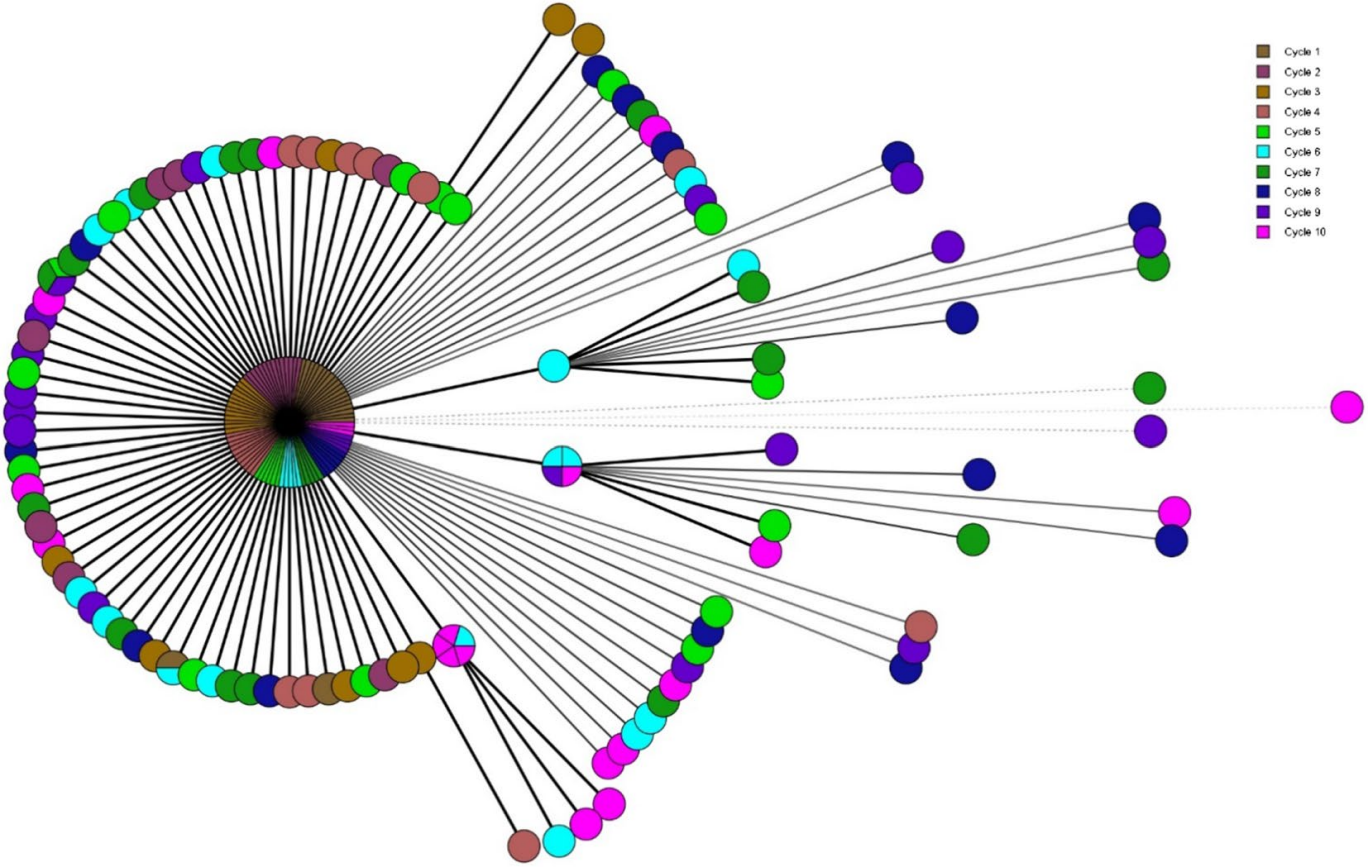
Maximum Likelihood Tree of SNP analysis of *S.* Agona. Each circle represents 1 isolate and the colors illustrate the heat treatment cycle (see Supplementary data 1).

The minimum spanning tree (MST) of the wgMLST analysis and ML tree of the SNP analysis for *S*. Agona is presented in Figs. 5 and 6, respectively. The isolates associated with the same treatment cycle are marked with the same color in both Figs. 5 and 6. The isolates from the initial cycle form the core of the MST and ML tree. As evidenced from the figures, the differences grew with increasing heat treatment cycles. The isolates did not group per cycle e.g. the isolates from the 10^th^ cycle are dispersed throughout the MST. A maximum of 28 allelic or 38 SNP differences were observed between 160 *S*. Agona isolates that were sequenced and analyzed. In Table 2, the maximum allelic and SNP differences noticed per cycle are mentioned. The number of differences accrued with every additional heat treatment cycle performed thus showing an increasing trend per cycle.

**Table 2:** Maximum differences per heat treatment cycle for *S.* Agona and *S*. Mbandaka.

Cycle	*S.* Agona Allele/SNP	*S*. Mbandaka Allele/SNP
1	9/6	2/6
2	11/12	2/4
3	12/16	4/5
4	14/17	5/8
5	22/23	4/9
6	20/23	4/10
7	19/25	8/19
8	21/27	8/11
9	26/32	8/11
10	28/38	8/10

**Figure 7:**
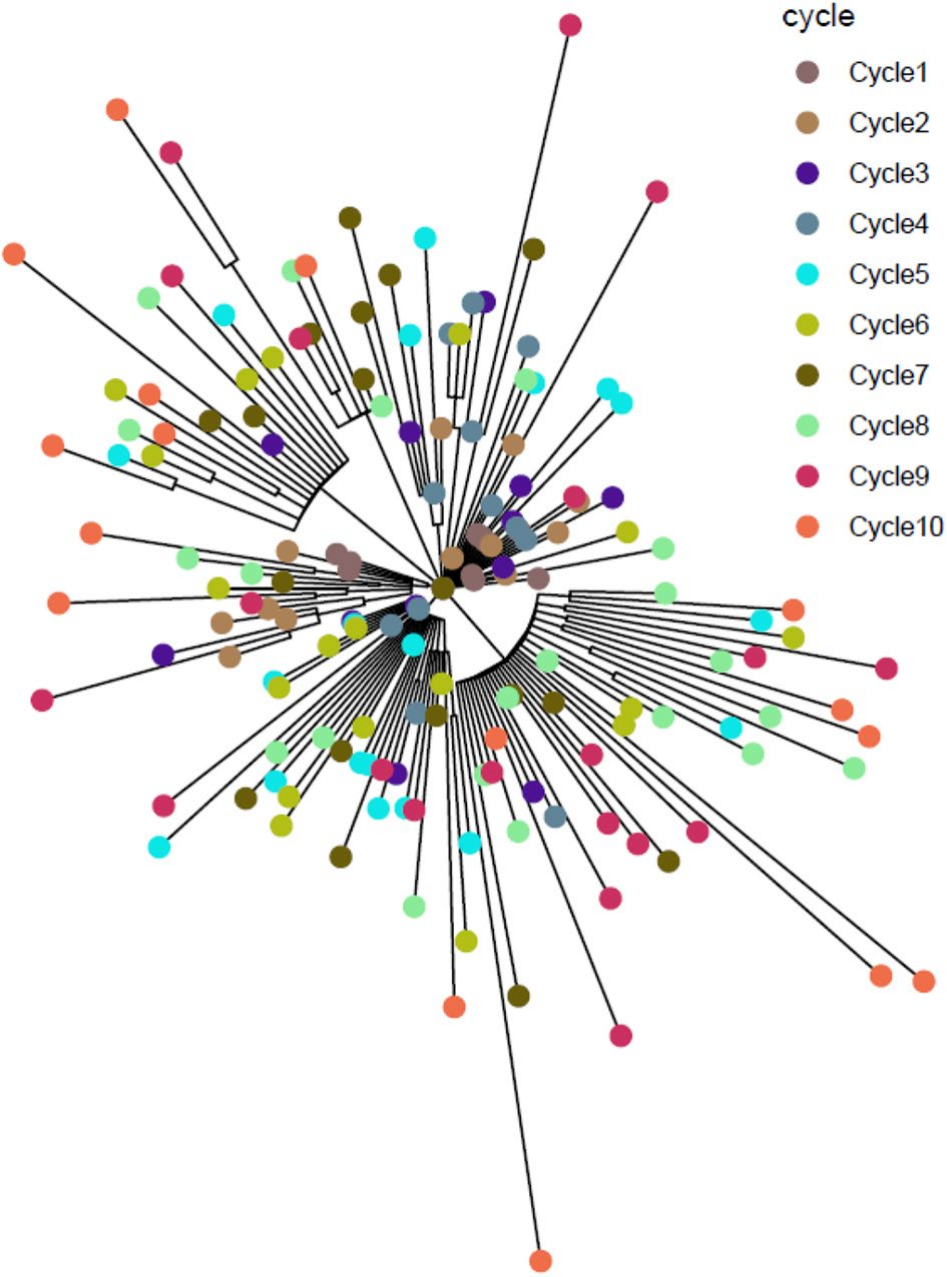
Minimum Spanning Tree of wgMLST analysis of *S.* Mbandaka. Each circle represents 1 isolate and the colors illustrate the heat treatment cycle.

**Figure 8:**
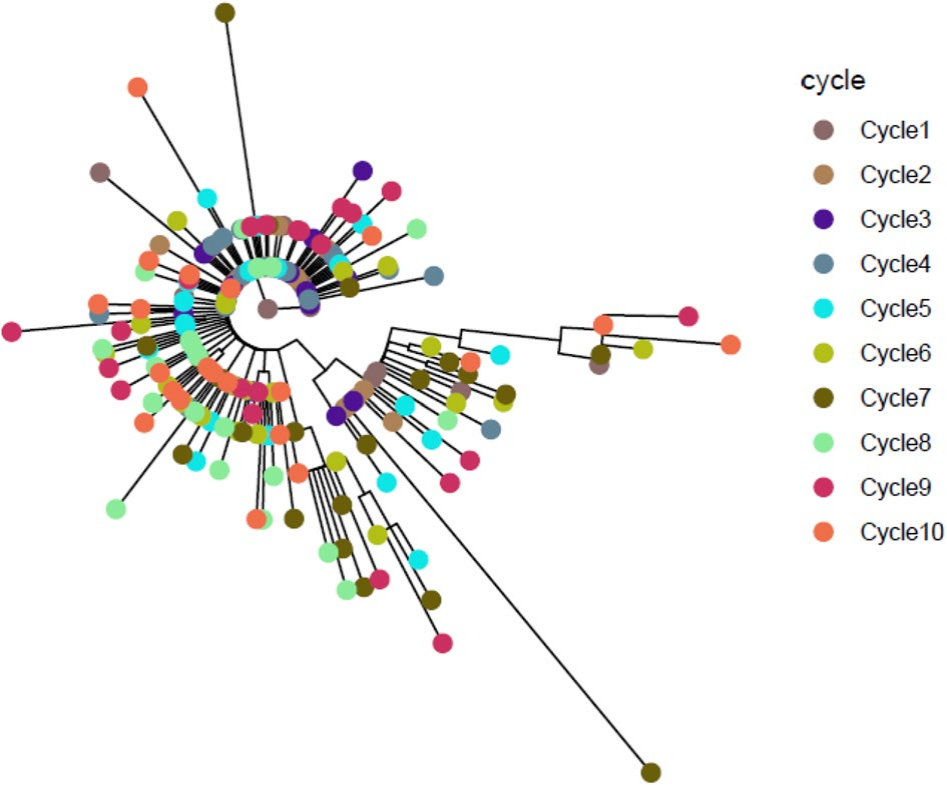
Maximum Likelihood Tree of SNP analysis of *S.* Mbandaka. Each circle represents 1 isolate and the colors illustrate the heat treatment cycle (see Supplementary data 2).

The MST obtained from wgMLST analysis of *S*. Mbandaka is shown in Figs. 7 and 8 represents the ML tree generated from SNP analysis. The maximum number of allelic and SNP differences is 8 and 19, respectively, among the 210 sequences obtained from *S*. Mbandaka isolates compared to 28 for *S*. Agona. The number of identical isolates (zero allelic/ SNP differences), represented by the core circle in Figs. 7 and 8, is higher when compared to S. Agona. Similar to *S.* Agona, an increasing trend of allelic differences was observed with the increasing number of treatment cycles, though it was less pronounced. A maximum of 19 SNP differences was noticed at cycle 7, and it decreased to 10 differences by cycle 10. Using wgMLST analysis, a maximum of 8 allelic differences was obtained after cycle 7 and it did not further increase.

Majority of the SNPs were unique in nature (91% for both serovars, 201/220 for *S.* Mbandaka and 722/794 SNPs for *S*. Agona) i.e. they were observed only once, suggesting that most of the mutations were most likely random in nature. In order to gain a preliminary understanding about the biological relevance of these SNPs, we investigated the SNPs that occurred minimum 10 times following exposure to stress conditions. Thus, for *S*. Mbandaka 5 SNP positions and for *S*. Agona 8 were selected for further investigation. The base change position and impact on the amino acid coded along with the gene or intergenic region implicated is presented in the supplementary Table S1. Four non-synonymous changes and one SNP in the intergenic region were observed for *S*. Mbandaka, whereas, *S*. Agona had 2 SNPs in the intergenic regions, 1 synonymous and 5 non-synonymous amino acid changes. A total of 2 out of 4 and 2 out of 5 SNPs creating non-synonymous changes in *S*. Mbandaka and *S*. Agona, respectively, were noticed in genes coding/ controlling flagellar functions.

## Discussion

Subtyping tools provide evidence or can bring new clues to direct the epidemiological investigation in an outbreak situation or for the root cause analysis of a pathogen contamination event in a food plant. These analytical results help to determine which isolates are “in” or “out” of the scope of an investigation. Traditional subtyping tools such as PFGE are being increasingly replaced by WGS as it provides a higher resolution to discriminate between pathogen isolates.

In 2017, a *S*. Agona outbreak caused by contaminated infant milk products among infants was identified in France^17^. The confirmed cases were analyzed by WGS and the SNP analysis revealed that the outbreak isolates clustered within a maximum of 26 SNPs^17^. In a different study from Germany, the potential similarity between a local *S.* Agona isolate from a feed sample to the French outbreak isolates was investigated^31^. Results indicated that the feed sample isolates differed by at least 40 SNPs in comparison to the French outbreak isolates and thus they were considered unrelated. However, the results also showed that an outbreak cluster linked to tea (2003) and a different cluster of coconut (1994) differed by only 21 SNPs to the isolate from the feed sample. It has been shown that fixed genetic cut-off values cannot be assigned due to the difference in evolution forces between organisms, the environmental exposure history of the organism and the context (e.g. duration of the outbreak, ability of an environment to support growth impacting generation time)^12,15^. In addition, the organisms can also be exposed to conditions causing sublethal stress injury during food processing that may induce genetic changes likely due to selection pressure. As low moisture and heat are two stress conditions that are present in a low moisture food producing facility, we questioned whether the exposure of *Salmonella,* known to be associated with low moisture foods, to these conditions could induce genetic changes. In this study, we therefore investigated the effect of heat stress on two *Salmonella* strains *S*. Agona and *S*. Mbandaka inoculated in a high fat, low water activity matrix.

The effect of the subsequent heat treatments upon the fitness of the strains was evaluated by the phenotypic characteristics such as inactivation by log reduction, lag time and sublethal injury. We observed a maximum of 2.1 log reduction in the matrix consisting of 25% fat and a water activity of less than 0.3. It has been shown that *Salmonella* is rapidly killed in liquid conditions but in matrices with high fat content and low water activity, the inactivation by heat is reduced^32^. Each heat treatment cycle resulted in > 98% levels of sublethal injury. The lag time did not show significant changes over the ten repeated heat cycles except for *S*. Mbandaka where a significant decrease in lag time was observed after one heat treatment. In conclusion, the phenotypic characteristics remained mostly unchanged over 10 repeated heat treatments in a low moisture, high fat matrix. This observation is in contrast to the study of Knöppel et al.^33^, who showed by serial passaging, and thus repeated exposure to, different growth media resulted in increased fitness. After 500–1000 generations in parallel cultures in 4 different media, 83 genetic changes consisting of amino acid substitution, deletion, duplication, frameshift mutation, intergenic mutation, frameshift reversion or pseudo reversion, non-sense mutation and synonymous mutation were identified for *S. enterica* LT2 by the authors.

On the genotypic level, genetic changes were observed for *S*. Agona and *S*. Mbandaka after the exposure to heat treatment in the low water activity, high fat matrix. It has been shown that *S*. Tennessee, Typhimurium and Enteritidis showed a maximum of 1 SNP difference after 10 sub-culturing events^24^. Maximum 3 substitutions after four passages in *S.* Montevideo was observed^34^. After 100 subcultures of *S*. Typhimurium and *S*. Newport, between 0 and 3 allelic differences were observed by wgMLST^35^. Thus, sub-culturing of different *Salmonella* serovars induced a maximum of 3 genetic changes. Therefore, we can conclude that significantly more genetic changes were introduced by exposure to repeated heat treatment, as observed in this study, than genetic changes that would have been expected by sub-culturing without stress. Additionally, an increasing number of allelic/SNP differences was observed after repeated exposure to heat. The number of genetic changes was higher for *S*. Agona compared to *S*. Mbandaka. The genetic diversity within *Salmonella* depends upon serovar e.g. *S*. Newport^36^ exhibit a large genetic diversity versus *S*. Enteritidis which has a low genetic diversity^19^. It should be noted that the laboratory simulations mimic stress conditions that might occur in an environmental production plant but does not reflect the amount of generations expected in these circumstances. After each heat treatment cycle, strains were incubated in non-selective medium for 24 h at 37 °C while in real production areas the chances of such occurrence are unlikely.

From the *S*. Agona MST, it seems that the randomly introduced genetic changes remains in the population and subsequent cycles introduce additional genetic changes in the isolates. As a result, the diversity of sequences increases with an increase in the number of heat treatment cycles. Similar observations, but to a lesser extent, were noticed for *S*. Mbandaka, i.e. the population with different genomic sequences was higher after cycle 10 compared to cycle 1. Overall, similar patterns were observed with the ML tree obtained for both serovars following the SNP analysis. In terms of genetic differences, SNP differences were almost always slightly higher than allelic distances but were comparable to each other for both serovars in different treatment cycles, except in cycle 1 for S. Agona. The higher number of differences noticed with the SNP analysis can be explained by the fact that about 95% of the genome is normally considered in the analysis, whereas the only coding genes are considered in wgMLST^11^. However, both these methods have been shown to provide concordant results for other organisms^12–14^ and the findings of our study confirms this observation for *Salmonella enterica*.

Although most mutations were unique and appeared to be random in nature, a few of them occurred several times. For example, a mutation in the gene coding for flagellar transcriptional regulator *flhD,* resulting in a non-synonymous amino acid change occurred in both serovars. FlhD protein is part of a transcriptional activator complex FlhD_4_C_2_, a master regulator of flagellar expression that then eventually allows the cell to be motile or non-motile based on the external and/ or internal stimuli^37^. In addition to FlhD, mutations in gene coding for flagellar motor switch protein (FliG) and flagellar basal body rod protein (FlgG) were also noticed. Physico-chemical environment, including temperature has been shown to affect flagellar expression and in general, the expression level is reduced with increased temperature. For example, Walker et al.^38^ have shown reduced flagellar expression in *S*. Enteritidis grown at 37 °C in comparison to 20 °C. Similarly, Sirsat et al.^39^ have shown the downregulation of flagellar genes of *S*. Typhimurium when exposed to sub-lethal thermal stress conditions through transcriptional profiling. In light of these reports and considering the fact that flagellar expression is a complex phenomenon that is intrinsically controlled at multiple levels^37^, we speculate that the genetic changes noticed in the genes related to flagellar expression would have resulted in its down regulation. In addition, mutations resulting in amino acid changes in other proteins such as NADP(H)-dependent aldo keto reductase, aspartate-semialdehyde dehydrogenase, CRISPR-associated helicase/ endonuclease Cas3 and glycosyl transferase (*gtr*) family I protein were also observed. Horizontally acquired *gtr* has been shown to contribute to the O-chain modification in the lipopolysaccharide component of outer membrane to enhance survival under adverse conditions in a host^40^. Thus, it is plausible that under exposure to stress conditions, the mutation induced in this gene would have resulted in the alteration of the composition of the O-chain to aid the survival of the organism. Additional research would be needed to confirm the precise role of the mutations observed in the genes and/ or intergenic regions.

## Conclusion

Exposure of *S*. Agona and *S*. Mbandaka to repeated heat treatments (90 °C for 5 min) in a low water activity and high fat matrix did not result in increased fitness of the strains. However genetic changes were introduced which resulted in a population of isolates with a maximum of 28 allelic differences (38 SNPs) for *S.* Agona and 8 allelic (19 SNPs) differences for *S.* Mbandaka. This phenomenon could also potentially contribute to the higher genetic differences that can be observed between isolates sampled over a rather short timeframe and this knowledge is key during the interpretation of WGS results in a source tracking investigation involving isolates that could have been exposed to heat and/ or dry stress conditions.

## Supplementary Information

Supplementary Information

## References

[1] Scallan E, Griffin PM, Angulo FJ, Tauxe RV, Hoekstra RM (2011). Foodborne illness acquired in the United States–unspecified agents. Emerg. Infect. Dis..

[2] Boore AL (2015). Salmonella enterica infections in the united states and assessment of coefficients of variation: a novel approach to identify epidemiologic characteristics of individual serotypes, 1996–2011. PLoS ONE.

[3] Pijnacker R (2019). An international outbreak of *Salmonella enterica* serotype Enteritidis linked to eggs from Poland: a microbiological and epidemiological study. Lancet Infect. Dis..

[4] Octavia S (2015). Delineating community outbreaks of Salmonella enterica serovar Typhimurium by use of whole-genome sequencing: insights into genomic variability within an outbreak. J. Clin. Microbiol..

[5] Thong KL (2002). Genetic diversity of clinical and environmental strains of Salmonella enterica serotype Weltevreden isolated in Malaysia. J. Clin. Microbiol..

[6] Finn S, Condell O, McClure P, Amezquita A, Fanning S (2013). Mechanisms of survival, responses and sources of Salmonella in low-moisture environments. Front. Microbiol..

[7] EFSA, Outcome of EC/EFSA questionnaire (2016) on used of Whole Genome Sequencing (WGS) for food- and waterborne pathogens isolated from animals, food, feed and related environmental samples in EU/EFTA countries. (2018).

[8] H. C. Bakker *et al.*, A whole-genome single nucleotide polymorphism-based approach to trace and identify outbreaks linked to a common Salmonella enterica subsp. enterica serovar Montevideo pulsed-field gel electrophoresis type. *Appl. Environ. Microbiol.* **77**, 8648–8655 (2011). 10.1128/AEM.06538-11 PMC3233099 22003026

[9] K. K. EFSA BIOHAZ Panel (EFSA Panel on Biological Hazards), Allende A, Alvarez-Ordo~nez A, Bolton D, Bover-Cid S, Che maly M, Davies R, De Cesare A, Hilbert F,Lindqvist R, Nauta M, Peixe L, Ru G, Simmons M, Skandamis P, Suffredini E, Jenkins C, Malorny B,Ribeiro Duar te AS, Torpdahl M, da Silva Felıcio MT, Guerra B, Rossi M and Herman L, Scientific Opinion on the whole genome sequencing and metagenomics for outbreak investigation, source attribution and risk assessment of food-borne microorganisms. *EFSA J.* **17**, e05596 (2019). 10.2903/j.efsa.2019.5898 PMC7008917 32626197

[10] Rouzeau-Szynalski K (2019). Whole genome sequencing used in an industrial context reveals a Salmonella laboratory cross-contamination. Int. J. Food Microbiol..

[11] Jagadeesan B (2019). The use of next generation sequencing for improving food safety: translation into practice. Food Microbiol..

[12] Jagadeesan B, Baert L, Wiedmann M, Orsi RH (2019). Comparative analysis of tools and approaches for source tracking listeria monocytogenes in a food facility using whole-genome sequence data. Front. Microbiol..

[13] Cunningham SA (2017). Comparison of whole-genome sequencing methods for analysis of three methicillin-resistant *Staphylococcus aureus* outbreaks. J. Clin. Microbiol..

[14] Katz LS (2017). A comparative analysis of the lyve-SET phylogenomics pipeline for genomic epidemiology of foodborne pathogens. Front. Microbiol..

[15] Pightling AW (2018). Interpreting whole-genome sequence analyses of foodborne bacteria for regulatory applications and outbreak investigations. Front. Microbiol..

[16] Wang YU (2018). Genetic diversity of salmonella and listeria isolates from food facilities. J. Food Prot..

[17] N. Jourdan-da Silva *et al.*, Ongoing nationwide outbreak of *Salmonella agona* associated with internationally distributed infant milk products, France, December 2017. *Euro. Surveill.* **23**, (2018). 10.2807/1560-7917.ES.2018.23.2.17-00852 PMC5770849 29338811

[18] Zhou Z (2013). Neutral genomic microevolution of a recently emerged pathogen *Salmonella enterica* serovar Agona. PLoS Genet.

[19] Deng X (2014). Genomic epidemiology of *Salmonella enterica* serotype Enteritidis based on population structure of prevalent lineages. Emerg. Infect. Dis..

[20] Koch J (2005). *Salmonella agona* outbreak from contaminated aniseed Germany. Emerg. Infect. Dis..

[21] Russo ET (2013). A recurrent, multistate outbreak of *Salmonella serotype* agona infections associated with dry, unsweetened cereal consumption, United States, 2008. J. Food Prot..

[22] EFSA, EU summary report on zoonoses, zoonotic agents and foodborne outbreaks 2016. *EFSA J.* **15**, 12 (2017). 10.2903/j.efsa.2017.5077 PMC7009962 32625371

[23] Hoszowski A, Zajac M, Lalak A, Przemyk P, Wasyl D (2016). Fifteen years of successful spread of *Salmonella enterica* serovar Mbandaka clone ST413 in Poland and its public health consequences. Ann. Agric. Environ. Med..

[24] Portmann AC (2018). A Validation approach of an end-to-end whole genome sequencing workflow for source tracking of *Listeria monocytogenes* and *Salmonella enterica*. Front. Microbiol..

[25] Rachon G, Penaloza W, Gibbs PA (2016). Inactivation of Salmonella, *Listeria monocytogenes* and *Enterococcus faecium* NRRL B-2354 in a selection of low moisture foods. Int. J. Food Microbiol..

[26] Gimonet J, Portmann AC, Fournier C, Baert L (2018). Optimization of subculture and DNA extraction steps within the whole genome sequencing workflow for source tracking of *Salmonella enterica* and *Listeria monocytogenes*. J. Microbiol. Methods.

[27] Davis S (2015). CFSAN SNP pipeline: an automated method for constructing snp matrices fromnext-generation sequence data. PeerJ Comput. Sci..

[28] D. J. Zwickl, Genetic algorithm approaches for the phylogenetic analysis of large biological sequence datasets under the maximum likelihood criterion. *Ph.D. dissertation, The University of Texas at Austin* (2006).

[29] Altschul SF, Gish W, Miller W, Myers EW, Lipman DJ (1990). Basic local alignment search tool. J. Mol. Biol..

[30] Carver TJ (2005). ACT: the Artemis comparison tool. Bioinformatics.

[31] Dangel A (2019). Genetic diversity and delineation of *Salmonella agona* outbreak strains by next generation sequencing, Bavaria, Germany, 1993 to 2018. Euro Surveill.

[32] Shachar D, Yaron S (2006). Heat tolerance of *Salmonella enterica* serovars agona, enteritidis, and typhimurium in peanut butter. J. Food Prot..

[33] Knoppel A (2018). Genetic adaptation to growth under laboratory conditions in *Escherichia coli* and *Salmonella enterica*. Front. Microbiol..

[34] Allard MW (2012). High resolution clustering of *Salmonella enterica* serovar Montevideo strains using a next-generation sequencing approach. BMC Genomics.

[35] Petronella N (2019). Changes detected in the genome sequences of *Escherichia coli*, *Listeria monocytogenes*, *Vibrio parahaemolyticus*, and *Salmonella enterica* after serial subculturing. Can. J. Microbiol..

[36] Zheng J (2017). Whole-genome comparative analysis of salmonella enterica serovar newport strains reveals lineage-specific divergence. Genome Biol. Evol..

[37] Soutourina OA, Bertin PN (2003). Regulation cascade of flagellar expression in Gram-negative bacteria. FEMS Microbiol. Rev..

[38] Walker SL, Sojka M, Dibb-Fuller M, Woodward MJ (1999). Effect of pH, temperature and surface contact on the elaboration of fimbriae and flagella by *Salmonella serotype* enteritidis. J. Med. Microbiol..

[39] Sirsat SA (2011). Effect of sublethal heat stress on *Salmonella typhimurium* virulence. J. Appl. Microbiol..

[40] Davies MR, Broadbent SE, Harris SR, Thomson NR, van der Woude MW (2013). Horizontally acquired glycosyltransferase operons drive salmonellae lipopolysaccharide diversity. PLoS Genet.

