# Supplementary material for: Genetic changes are introduced by repeated exposure of Salmonella spiked in low water activity and high fat matrix to heat
Source: Sci Rep. 2021 Apr 14;11:8144. doi: 10.1038/s41598-021-87330-8 (PMC8046991; doi:10.1038/s41598-021-87330-8)
Supplement: Supplementary file 1 — Supplementary Information [file 41598_2021_87330_MOESM1_ESM.docx]

**Supplementary Material**

**Genetic changes are introduced by repeated exposure of *Salmonella* spiked in low water activity and high fat matrix to heat**

Leen Baert^1^, Johan Gimonet^1^, Caroline Barretto^1^, Coralie Fournier^2^, Balamurugan Jagadeesan^1^

^1^Nestlé Research. Vers-Chez-les-Blanc, 1000 Lausanne 26, Switzerland.

Correspondence and requests for materials should be addressed to L.B. (email: [Leen.Baert@rdls.nestle,com](mailto:Leen.Baert@rdls.nestle,com))

^2^Nestlé Research. EPFL Innovation Park, 1015 Lausanne, Switzerland.

**Table S1. Impact of SNPs in the genes/ intergenic regions of *S*. Mbandaka and *S*. Agona**

| **Organism** | **SNP position (based on reference genome)** | **Coding/ Non coding region** | **Base change** | **Amino acid change** | **Frequency of occurrence** |
| --- | --- | --- | --- | --- | --- |
| *S*. Mbandaka | 2322693 | Noncoding intergenic region before EAL domain containing protein | C 🡪 A | Not applicable | 14 |
|  | 2082918 | Coding, Flagellar transcriptional regulator, FlhD | T 🡪 G | M 🡪 R | 11 |
|  | 2047064 | Coding, Flagellar motor switch protein, FliG | C 🡪 A | E 🡪 D | 13 |
|  | 930237 | NADP(H)-dependent aldo keto reductase | G 🡪 C | R 🡪 P | 10 |
|  | 1869755 | Glycosyl transferase family I protein | T 🡪 G | Q 🡪 K | 60 |
| *S*. Agona | 84387 | Noncoding, intergenic region before gene coding for phenylalanine transporter | C 🡪 T | Not applicable | 142 |
|  | 9901 | Coding, Inorganic phosphatase | A 🡪 C | Q 🡪 P | 38 |
|  | 240128 | Coding, Flagellar transcriptional regulator FlhD | A 🡪 C | L 🡪 P | 38 |
|  | 42961 | Noncoding, intergenic region before gene coding for toxin-antitoxin system ParD family antitoxin | A 🡪 C | Not applicable | 38 |
|  | 393760 | Coding, Flagellar basal body rod protein FlgG | A 🡪 C | E 🡪 A | 36 |
|  | 191927 | Coding, DUF4424 family protein | A 🡪 C | K 🡪 Q | 16 |
|  | 55354 | Coding, Aspartate-semialdehyde dehydrogenase | A 🡪 C | I 🡪 L | 16 |
|  | 18726 | Coding, CRISPR-associated helicase/ endonuclease Cas3 | T 🡪 G | G 🡪 G | 16 |
